# How Important Is Eating Rate in the Physiological Response to Food Intake, Control of Body Weight, and Glycemia?

**DOI:** 10.3390/nu12061734

**Published:** 2020-06-10

**Authors:** Georgia Argyrakopoulou, Stamatia Simati, George Dimitriadis, Alexander Kokkinos

**Affiliations:** 1 Diabetes Unit, Athens Medical Center, 151 25 Marousi, Greece; 2 First Department of Propaedeutic Internal Medicine, Medical School, National and Kapodistrian University of Athens, Laiko Hospital, 115 27 Athens, Greece; simatistemi@gmail.com (S.S.); rjd@otenet.gr (A.K.); 3 Medical School, National and Kapodistrian University of Athens, 115 27 Athens, Greece; gdimitr@med.uoa.gr

**Keywords:** eating rate, body weight, glycemia, energy intake, hunger, satiety

## Abstract

The link between eating rate and energy intake has long been a matter of extensive research. A better understanding of the effect of food intake speed on body weight and glycemia in the long term could serve as a means to prevent weight gain and/or dysglycemia. Whether a fast eating rate plays an important role in increased energy intake and body weight depends on various factors related to the studied food such as texture, viscosity and taste, but seems to be also influenced by the habitual characteristics of the studied subjects as well. Hunger and satiety quantified via test meals in acute experiments with subsequent energy intake measurements and their association with anorexigenic and orexigenic regulating peptides provide further insight to the complicated pathogenesis of obesity. The present review examines data from the abundant literature on the subject of eating rate, and highlights the main findings in people with normal weight, obesity, and type 2 diabetes, with the aim of clarifying the association between rate of food intake and hunger, satiety, glycemia, and energy intake in the short and long term.

## 1. Introduction

Obesity is linked to several metabolic disturbances such as type 2 diabetes mellitus (T2DM), atherosclerosis and heart disease [1]. Extensive research has been conducted in order to clarify if simple advice such as eating at a slower rate or eating foods of different texture could be effective in curtailing energy intake and weight gain in the long term as well as if such strategies could play a role in glycemia. 

The association of obesity with a fast eating rate has been shown both in normoglycemic as well as in subjects with T2DM [2,3,4,5]. In addition, multiple studies have shown that engaging in a fast eating rate results in increased BMI [6]. A retrospective eight-year study compared three groups of male workers according to their speed of eating and found that the fast-eating group had a higher mean weight gain [7]. Accordingly, in a three years follow-up study of normal-weight individuals, the risk of gaining weight was increased in those that reported eating faster [8].

Eating rate can be measured in g or kcal of food consumed per min. Satiety, or intermeal satiety (i.e., the feeling of fullness throughout the intermeal interval) and hunger (i.e., the conscious sensation reflecting a mental urge to eat) are usually measured via visual analog scales (VAS) and are self-reported, and so remain subjective, although they are generally methodologically accepted [9,10]. It is unclear if the effect of test meals on hunger and satiety in acute experiments and subsequent energy intake apply in everyday life. Additionally, different study groups, i.e., healthy individuals vs. subjects with diabetes could respond differently, not to mention different BMI groups. Constant emerging data concerning anorexigenic and orexigenic peptides make their potential association to corresponding alterations in hunger and satiety a promising field. 

Eating rate could be attributed to a person’s behavioral characteristics as well, as it seems that subjects who eat a certain product faster would also consume another product at a faster rate [11]. When monozygotic twin pairs were compared to dizygotic twins, a higher correlation of eating rate was shown, perhaps pointing to a genetic contribution to eating rate [12].

Most of the relevant literature converges to support the notion that eating at a faster rate leads to increased energy intake [13]. Less evidence points towards increased satiety after manipulated eating rate [13].

The present review aims to untangle the labyrinthine landscape in the field of eating speed manipulation, which is comprised of very heterogeneous interventions, by attempting to interpret the relevant literature towards potential unifying concepts. It also presents a collection of practical devices which are being tested towards the end of food intake manipulation as a means of controlling body weight.

Herein, we present the most representative studies examining eating rate, food texture/density and masticatory cycles and divide them depending on the subject groups (healthy, obese and individuals with diabetes). Two authors independently conducted an online PubMed search using relevant key words; eating rate, eating speed, fast eating, rapid eating, slow eating combined, with additional key words such as body mass index, body weight, and obesity, diabetes, glycemia, glucose. Studies in patients with eating disorders (binge eating, bulimia or anorexia) were excluded as well as studies with no clear manipulation of eating rate, those concerning children/adolescents and studies with language restrictions. Lastly, we included some novel studies concerning devices that manipulate eating rate.

## 2. Studies Concerning Healthy Individuals

### i. Manipulating Eating Rate

**Figure 1:**
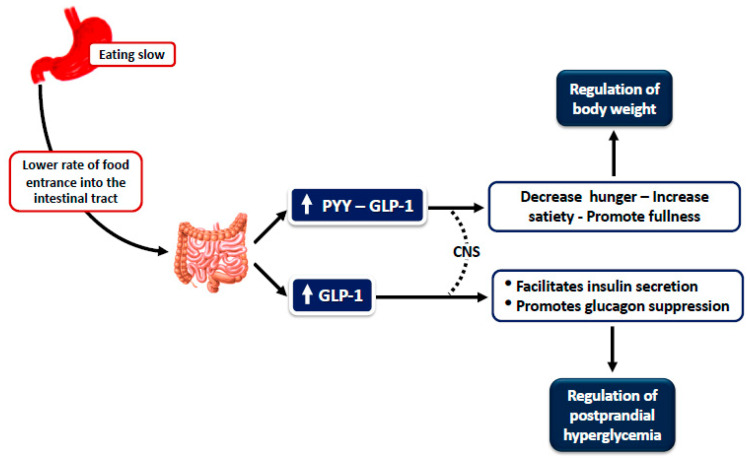
Schematic presentation of gut hormone responses and clinical outcomes in healthy individuals eating at a slow rate [15].

Andrade et al. examined healthy women (including normal weight, overweight and obese) and found that satiety, using VAS, was significantly higher and energy intake was lower when a slower eating rate was applied, whereas hunger and desire to eat did not differ at the end of the meal [14]. Our group examined the effect of eating rate on the postprandial levels of appetite-regulating hormones in healthy individuals (both normal weight and overweight) and produced a hypothesis that suggests that eating at a slower rate is associated with increased satiety, higher plasma levels of anorexigenic peptides PYY (peptide tyrosine tyrosine) and GLP-1 (glucagon like peptide 1) and lower plasma levels of the orexigenic peptide ghrelin [15]. Subjects consumed an identical meal at two separate sessions of different durations, namely, 5 min versus 30 min. We found that postprandial responses of plasma PYY and GLP-1 were higher after the 30- vs. the 5-min meal, but postprandial levels of ghrelin did not differ significantly (whilst there was a trend for lower ghrelin levels at the 120-min time point for the 30-min meal) (Figure 1). Thus, we speculated that eating quickly elicits weaker anorexigenic gut hormone responses. However, a potent effect on ghrelin was not shown. Consistent with other studies, glycemia and insulin levels were not different between the two meals. However, we did not measure energy intake at the subsequent meal, or the time elapsed until initiation of the next meal; therefore, whether the documented effects influence satiety remains unclear [15]. 

One would wonder if food intake and, subsequently, weight management could be manipulated via all aforementioned parameters or if behavioral patterns are equally or even more important in that aspect. A potential hereditary influence in monozygotic twins has already been mentioned [12]. The role of individual eating patterns on food intake and satiety, when the rate of eating diverts from the subject’s habituated rate, i.e., examining the effect of an increase or decrease in eating rate, as well as the effect of taking a break while eating in so-called linear and decelerated female eaters, was studied [16]. Interrupting the meal has been thought to reduce eating rate and food intake. Women eating at a decelerated rate presented with a difficulty increasing their rate of eating and under control conditions reported higher satiety compared to the linear eaters. They also ate significantly less when the meal was short and when eating rate was increased, while the opposite was found for linear eaters. No effect on food intake was found with a decreased eating rate or meal interruption on decelerated eaters, while linear eaters ate significantly more food when the meal was interrupted, but less food when eating rate was decreased [16].

The question of whether eating rate is also influenced by ongoing perceptual estimates of the volume of food remaining and by a corresponding adjustment of food intake during a meal, has also been examined [17]. Subjects were “tricked” into eating more or less than what appeared and were unaware that their portion size had been manipulated. Participants who saw 300 ML but actually consumed 500 ML ate at a faster rate than participants who saw 500 ML but consumed 300 ml. When food disappeared faster or slower than anticipated, subjects adjusted their rate of eating accordingly. Eating rate may also be controlled via visual feedback and is not considered a simple reflexive response to orosensory stimulation. Irrespective of food type, participants reported greater fullness at the end of the meal if they had consumed the 500 ML portion compared to participants who had eaten the 300 ML portion [17].

The association between eating rate and basal metabolic rate (BMR) and its association with energy intake requirements has revealed interesting results [18]. A possible driving force of energy intake could be an individual’s energy requirements, estimated via measurement of BMR. Basal metabolic rate was positively associated with eating rate, independently of BMI. Thus, one could possibly attribute faster eating rates with subsequent higher food intake to adaptive behaviors in order to meet higher energy requirements [18]. Furthermore, it seems that eating rate is relatively stable within an individual and is not dependent on meal palatability, sex, body composition and reported appetite. That is, the recorded fast eating rate at a meal predicts a similar rate along with increased energy intake at subsequent meals [19]. 

Normal-weight volunteers were examined while consuming a meal in either 6 min or 24 min [20]. Slower eating suppressed ghrelin to a greater extent, and there seemed to be a strong correlation between postmeal ghrelin and post-test ad libitum meal intake, i.e., individuals eating at a slower rate consumed a smaller quantity of the subsequent ad libitum meal, and reported feeling fuller from the 30-min time point and for the rest of the one-hour study. On the contrary, the normal eating rate group (6 min) showed a greater PYY response compared to the slow rate group and reported greater satisfaction from the meal. Patients underwent an functional magnetic resonance imaging (fMRI) test 2-h postmeal while undergoing a memory task concerning the meal. The slower eating subgroup reported more accurate portion size memory with a linear relationship between time taken to make portion size decisions and the BOLD (blood-oxygen-level dependent) response in satiety and reward brain regions [20]. Detailed information regarding the aforementioned studies are presented in Table 1. 

### ii. Manipulating Food Texture

Texture of food, on the other hand, seems to play a significant role in eating rate as well, which subsequently influences food intake (Τable 1). Forde et al. examined healthy, normal-weight individuals and concluded that food of softer texture and high savory taste intensity leads to increased energy intake, increasing the average eating rate in the softer texture food by approximately 20% [21]. In the same context, the difference in energy intake (in kcal and g) after eating different food textures showed that harder foods led to a 16% lower intake compared to softer foods [22]. The overall eating rate of hard texture food was 32% lower compared to that of softer food. However, this did not decrease energy intake when subjects were introduced to a meal five hours later, thereby questioning the effect of food texture on long-term weight management [22].

The combined impact of eating rate and meal density was studied in 20 healthy individuals eating at two different speeds (fast vs. slow; 15 min longer when slower rate was applied) as well as using two different energy density meals [23]. Energy density was defined as the metabolizable energy per gram of food (kcal/g). Energy intake was higher when participants ate at a faster rate, but this effect applied only to high density food, whereas the effect of energy density οn energy intake was observed at both eating rates, but was potentiated by 43% when faster eating rates were implemented on high density meals. The postprandial area under the curve (AUC) for insulin, PYY and GLP-1 were higher during the fast and high energy density trials, but appetite remained relatively unaffected, as did glucose and ghrelin levels. The authors concluded that a faster eating rate had a greater effect on energy intake when a high energy density compared to a low energy density was consumed and, thus, it seems that adopting an eating pattern that includes frequent consumption of high energy dense food at a fast rate may eventually promote overeating [23]. 

Foods differing in viscosity (liquid vs. semiliquid) lead to differences in ad libitum intake and meal termination, producing differences in eating rate [24]. Food intake seems to increase with decreasing viscosity and the mechanisms involved are, at least to a certain extent, the shorter sensory exposure time and transit time food spends in the oral cavity. This effect, tested both in the real world as well as in the laboratory setting, was not due to differences in energy, macronutrient content or energy density, as they were all identical (and thus potential confounders in liquid–solid differences in satiety were controlled) [24]. The eating rate of the liquid product was significantly higher than the eating rate of the semisolid product, suggesting that a liquid is, as expected, eaten at a much higher rate and does not stay long in the oral cavity compared to the solid product. The time a product actually stays in the oral cavity could be an important parameter explaining the differences in satiety responses between liquids and solids, since the exposure time to sensory receptors in the oral cavity is longer for taste, smell, etc. Furthermore, in this study there were no differences in satiety after ad libitum intake—despite the differences in food intake—supporting the fact that subjects did not feel less full after the larger consumption of the liquid product. Perhaps energy intake and satiety do not correlate well in both liquids and solids. Interestingly, when the same amounts of calories are consumed, the subjective feelings of satiety are different and when the subjective feelings of satiety are the same, the amount of calories consumed is different. Multiple studies have shown that hunger and satiety are not always correlated to energy intake and certainly, in that aspect, do not foresee weight gain [24]. The effect of lower viscosity (produced by the modification of b-glucan content) produced a greater decrease in postprandial ghrelin and a greater postprandial increase in satiety, plasma glucose, insulin, cholecystokinin, PYY, and GLP-1, accentuating the importance of rheological properties of food in normal-weight subjects [25]. The high-viscosity vs. the low-viscosity oat bran beverage induced smaller postprandial glucose and insulin responses, consistent with delayed gastric emptying [25].

Different macronutrient consumption tendencies and their particular association with food intake and ingestion time have been measured in normal-weight participants [26]. Marked differences were observed in eating rate between foods, i.e., even within a food category such as solid foods, eating rate differed up to 30 times. Eating rate was positively associated with energy intake and inversely associated with energy density. For every 10 g/min increase in eating rate, energy intake increased by 1%. Carbohydrate, protein, and fiber content were inversely associated with eating rate in contrast to fat, which showed no association [26]. This could relate to the low water content or the increased density that would necessitate increased mastication. Given that fat is a fundamental determinant of energy density, its nonassociation with eating rate may imply that fat has minimal inhibitory effect on eating rate, as seen in its effect to elicit satiation [27].

### iii. Manipulating Masticatory Cycles

Another intriguing aspect of food intake regulation is the masticatory cycles a person undergoes before swallowing a certain food (Τable 1). Energy intake was assessed using an ad libitum specific test meal high in carbohydrates, after instructing the subjects to eat it in two separate sessions, where the number of masticatory cycles as well as the duration of the meal were different [28]. Increasing masticatory cycles before swallowing increases satiety, as measured by subjective appetite questionnaires, but does not lead to a difference in food intake. There was a trend towards an effect of masticatory cycles on ghrelin, with lower ghrelin following the higher number of cycles, as well as higher plasma glucose, insulin, GIP and CCK concentrations. Ghrelin and CCK did not seem to correlate with increased satiety. The authors attributed the findings, among others, to the property of decreasing the particles’ size, subsequently increasing the bioavailability of nutrients [28].

In lieu of commonly used methods to quantify hunger and energy intake, Mattes et al. showed that in normal-weight participants, hunger ratings are not a valid index of energy intake computed from food records or number of eating occurrences, since participants often ate when hunger ratings were low. Nevertheless, eating when not hungry occurred less often than not eating when hungry [29]. 

## 3. Studies Concerning Patients with Overweight/Obesity 

Numerous studies have shown that among the multifactorial nature of obesity, the detrimental effect of genes prevails among all [30]. Nevertheless, several papers have dealt with the impact of manipulating environmental factors and still the question of whether all findings could apply in the long term remains. By the term satiation, we describe the process taking place during a meal that leads to the termination of eating, consequently affecting and controlling energy intake (intrameal satiety). Satiety, on the other hand, describes the inhibition of further eating, decline of feelings of hunger, increase in fullness after a meal has finished (postingestive satiety or intermeal satiety). It could, therefore, be determined by the ad libitum energy intake during the next meal [10]. Eating pattern differences between subjects include bite size, eating rate, masticatory cycles, speed, etc. [31]. A number of studies (presented in Table 2) have sought these differences between obese and normal-weight individuals, but the literature has not yet yielded conclusive results. It would be very significant if people with obesity could benefit from small everyday interventions, once proven that they might confer positive results in weight management.

### i. Manipulating Eating Rate

Rapid eating does seem to be more frequent among overweight/obese patients. When given the same advice regarding eating rate, overweight/obese individuals ate at a faster rate compared to a normal-weight group [32]. Normal-weight and obese volunteers were studied after consumption of a meal at three different eating rates (7-, 14- and 28-min duration) evaluating the effect of eating rate on postprandial fullness and associated postprandial hormonal responses (PP, GLP-1, PYY, cholecystokinin, leptin and neuropeptide Y) and energy intake during the subsequent ad libitum meal [9]. Postprandial glucose and insulin responses were not affected by eating rate, and although eating at a faster rate altered peak PP concentrations and periprandial CCK response when compared to the moderate and slow eating rate, they were not different between meals, indicating no persisting effect. No effect was shown on the energy intake of the subsequent ad libitum meal at any eating rate. These findings imply that eating rate does not influence satiety or fullness despite a weak effect on the periprandial hormonal response [9]. This is in contrast to the findings of our study, in which postprandial PYY and GLP-1 were decreased when increasing the eating rate of a test meal [15]. Comparing the effect of eating rate on energy intake between normal-weight and overweight/obese subjects during an ad libitum meal, energy intake differed in the normal-weight but not in the overweight/obese subjects [33]. Martin et al. examined overweight and obese men and women and showed that a slow eating rate also decreased food intake [34]. However, this was shown only in men. Among other explanations for this discrepancy, authors hypothesized that men eat faster than women, and so it would be possible that subgroups of women who do eat at a faster rate could be more sensitive to different eating rates. Nevertheless, appetite was affected by the decelerated eating pattern during the combined-rate meal (a meal starting at baseline speed followed by a 50% slower eating rate) [34]. When a bite-counter device was used to manipulate eating rate, a decrease in energy intake accompanied slow bite rate, but only in those who habitually ate larger quantities of food during a meal (more than 400 kcal) [35].

### ii. Manipulating Masticatory Cycles

Smit et al. showed that subjects reduced their energy intake by 12% when chewing a standard meal 35 vs. 10 times per mouthful [36]. Although instructing participants to chew more ultimately led to faster chewing, it still resulted in a longer meal duration (a near 100% increase). Postprandial fullness ratings, however, did not differ. When habitual chewing pattern was assessed and compared between normal-weight and obese participants, no difference was found [36]. 

In an older publication, the hypothesis that decreasing ingestion rate would lead to smaller energy consumption was not confirmed and there were no significant differences between normal- weight and overweight/obese subjects [37]. Apparently, there are consistent individual differences in eating behavior that characterize faster compared to slower eaters, i.e., faster eaters were more sensitive to variations in bite size and to the texture of the food but, as aforementioned, these were not related to the amount of energy intake [37].

A study in severely/morbidly obese patients assessed the relationship between eating rate and parameters of eating behavior [38]. Among others, rapid eating was considered a significant risk factor for complications after bariatric surgery [38]. Female patients suffering from severe or morbid obesity, most of which were awaiting bariatric surgery, took a self-administered questionnaire that explored eating rate, degree of chewing, signs of prandial overeating and scores of emotionality, externality and restrained eating [39]. Fifty percent of the examined patients reported rapid eating, which was also associated with the feeling of having eaten too much. Additionally, there was an inverse relationship between eating rate and degree of chewing [39].

## 4. Studies Concerning Subjects with Diabetes Mellitus

In a multicenter center study examining subjects with T2DM using a self-reported questionnaire, BMI seemed to increase with increases in the rate of eating [40]. A high prevalence of rapid eaters was noted (61.5%), compared with studies in healthy controls, i.e., 36.5–50.8% [3,4,40]. To our knowledge, comparison studies have not been conducted between subjects with and without diabetes regarding their eating rate. 

Type 2 diabetic individuals seem to be more resistant to weight loss in comparison to nondiabetic groups, an observation which is not fully understood [41]. Based on the studies supporting that the incretin effect is blunted in obese subjects with T2DM, our group opted to study the effect of eating rate on hunger, satiety, and on the enteroendocrine hormone axis in overweight/obese patients with type 2 diabetes mellitus using a standard test meal of 300 ML of ice-cream consumed at two different rates [42,43]. Postprandial levels of insulin and glucose were not affected by eating rate, nor were ghrelin, PYY, and GLP-1, but slow spaced eating did result in a decrease in hunger and an increase in fullness [42]. 

Subjects with T2DM or hyperlipidemia were examined using a questionnaire assessing their eating rate [5]. Fast eating male patients displayed a higher BMI, but that did not apply to females, perhaps due to their smaller number. However, subjects were not analyzed separately, providing confounding factors in the interpretation of the results [5]. Details with reference to the aforementioned studies are presented in Table 3.

## 5. Devices Manipulating Eating Rate

### i. Noninvasive Oral Devices

**Figure 2:**
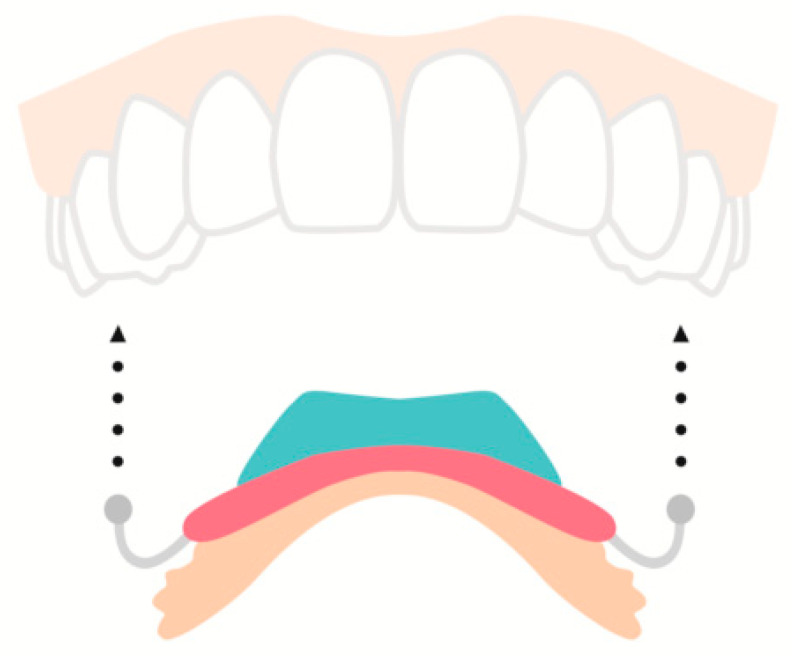
This oral device is placed in the upper palatal space and is secured by metal clasps right before initiation of eating. A microchip (shown in blue) records the device’s temperature in order to monitor the user’s compliance. It is designed to decelerate eating rate by decreasing oral volume and the size of each bite [47].

An oral device, custom-made for each individual, designed to decelerate eating rate by decreasing oral volume and bite size was studied in obese/overweight individuals in a 4-month open label trial (Figure 2) [47]. The device is placed in the upper palatal space and secured by metal clasps around the teeth before initiation of the meal and is removed after termination. Participants exhibited a 5.2% weight loss while using the aforementioned device accompanied by a hypocaloric diet. Participants reported eating slower even when the device was not used [47]. 

Intraoral splints for both the upper and lower jaw, extending about 3 mm over the premolars and molars, reduce oral capacity by 25% and alter eating behavior without preventing users from eating [48]. At a 12-month follow-up in a study where the device was used for a total of 4 to 8 weeks, all participants exhibited weight loss of up to 5% and an impressive 67% experienced 10% weight loss [48]. 

**Figure 3:**
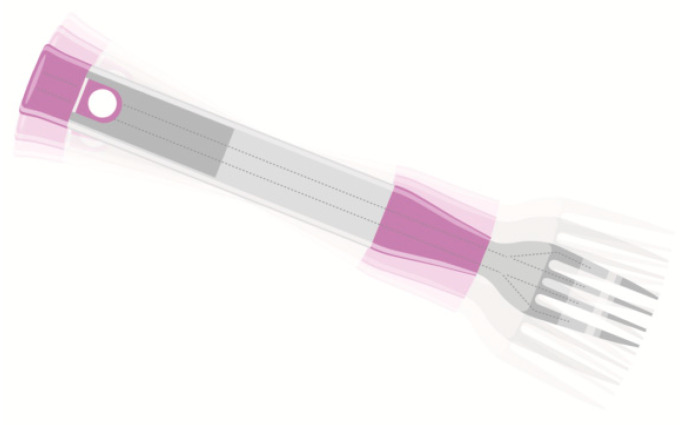
A smart fork that helps the user to decrease eating rate by calculating eating speed and meal duration. A red-light indication and a vibration appear when eating rate is accelerated [50].

Furthermore, objects of everyday use, like cutlery, could be potentially modified in order to decrease eating rate, i.e., smaller spoons resulted in a decrease in ad libitum food intake by 8%, decreasing both mean bite size and eating rate [49]. A smart fork has been designed in order to assist the user to maintain a slow eating rate by determining meal duration and calculating total number of bites (Figure 3) [50]. The device vibrates and a red-light indication appears every time eating rate is accelerated (more than one bite per 10 s) [50]. A three-armed parallel randomized controlled trial consisting of a group using the fork with vibrating feedback, a second group using the fork with access to online data (for eating rate and success ratio feedback), and a third group using the fork with no feedback resulted in weight loss in the intervention groups [51]. At a follow-up, participants maintained a decreased eating rate by longer spacing between bites and a lower bite rate [51]. 

**Figure 4:**
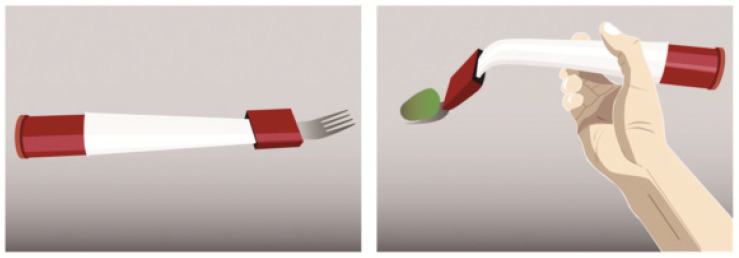
A pneumatic fork that changes its body shape by inflating and deflating through a small pump and valve depending on the detected eating rate [52].

A pneumatic fork that changes its body shape by inflating and deflating through a small pump and valve has also been used for the detection of accelerated eating rate (Figure 4) [52]. It bends when deflated, making it unable to eat with [52].

### ii. Wearable Devices

**Figure 5:**
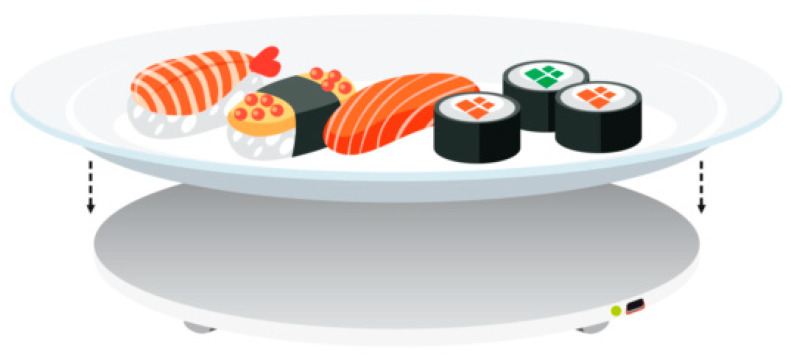
A custom-made electronic scale that is connected via bluetooth with a smartphone application measuring the reduction of the food placed on the scale along with self-recording of hunger and fullness [54].

The detection and monitoring of eating habits can also be conducted using smart eyeglasses with integrated electromyography electrodes on each side that provide skin contact with the ears [53]. Via high detection of chewing and eating events (ca 80%), the device could possibly contribute to dietary monitoring [53]. A device providing visual feedback via an application on a smartphone attempts to manage body weight by manipulation of eating rate [54]. An electronic scale is connected via Bluetooth with the smartphone and measures the gradual reduction in food on the plate. Self-recording of hunger and fullness is also available on the screen (Figure 5) [54].

## 6. Effect of Eating Rate on Glycemic Response 

Studies manipulating eating rate conducted directly to evaluate its effect on glycemia are scarce. Different eating methods can affect eating rate which may in turn influence postprandial glucose responses. Eating methods (spoon, chopsticks and fingers), and a mastication manipulation method as potential means of lowering glycemic response, taking into consideration that the amount of food provided per mouthful and chewing time differs between eating methods, have been studied [44]. Eating with chopsticks resulted in decreased postprandial glucose response, higher chewing rate (chews per mouthful divided by chewing time), smaller bite size, smaller number of chews per mouthful and a decreased eating rate [44]. Healthy participants’ glycemic response (via finger-prick) was studied while consuming large vs. small rice particles [45]. Gastric emptying (using the sodium [^13^C] acetate breath test) was also assessed. Small particles elicited a significantly greater glycemic and insulin response compared to large particles and induced faster gastric emptying [45]. Modifying the mastication rate could alter the glycemic index of rice, i.e., less mastication cycles induced significantly lower glycemic response and lower glycemic index [46].

Glycemia (assessed with HbA1c) showed no association with increased eating rate reported via a self-reported questionnaire in subjects with type 2 diabetes, [40]. However, this would be expected, presumably via an increase in postprandial hyperglycemia [55]. Increased eating rates may induce a faster entrance of glucose into the circulation, requiring an immediate response from β-cells. In type 2 diabetes, the delay of insulin secretion after a meal is a major pathophysiological feature of postprandial hyperglycemia: restoration of early insulin secretion in subjects with type 2 diabetes after a mixed meal resulted in adequate suppression of endogenous lipolysis and lower plasma glucose levels in the postprandial period [56]. Moreover, in subjects with diabetes, the delay in gastric emptying and intestinal glucose absorption after a meal by α-glucosidase inhibitors or somatostatin, improved time differences between postprandial plasma glucose and insulin increases, thus leading to lower postprandial hyperglycemia [57,58,59].

Regarding the effect of eating rate on insulin resistance, a significant progressive increase in homeostatic model assessment of insulin resistance (HOMA-IR) was found with increases in relative eating rate in healthy middle-aged normal-weight individuals, suggesting that eating rate is independently associated with insulin resistance [60]. These observations could be explained by the rapid entrance of glucose into the circulation in the beginning of the meal, which may aggravate postprandial hyperinsulinemia, leading in turn to increased fluctuations of circulating blood glucose levels [56,61].

## 7. Conclusions

Hitherto, a substantial amount of studies has pointed to the direction that eating rate is an important factor influencing energy intake in acute settings, such that those who eat quickly seem to eat more compared to those who eat at a slower pace, all within a meal. This tendency increases satiation, but in most circumstances, it does not alter satiety responses and energy intake in subsequent meals, nor does it increase the intermeal interval. Thus, it would not translate into measurable behavioral changes affecting weight gain. Relevant studies show dissimilar results. The question of whether eating quickly could be used as a predictor of the risk of gaining weight in the long term remains. In addition, whether eating rate acutely or chronically affects glycemia remains a largely unanswered question. Food texture and hereditary/habitual characteristics along with eating rate are important features that affect food intake, eliciting a different response depending on the setting and the population studied. Neuroendocrine gut hormone response studies, assessing ad libitum energy intake at different eating rates could be useful in order to quantify the basis of both satiation and satiety produced by different patterns of eating. 

## Figures and Tables

**Table 1:** Studies examining the effect of manipulating eating rate, food texture, and mastication speed in healthy individuals.

First Author, Year, (Ref)	Participants	Study Design	Eating Rate Manipulation	Ad Libitum Meal after Standardized Meal	Hunger Measurements	Biochemical Profile and Hormones Response	Results
Zijlstra 2010 [11]	N = 106	Ad libitum meals (seven) in a real-life setting (cinema)	Test meal: luncheon meat, vegetarian meat replacer and chewy candy	no	Type: VAS	Not measured	Fast condition
	Healthy		Soft (fast condition) and hard (slow condition) version		Time: at the beginning and end of the movie		ER: 25 ± 13 g/min
	45 males						Intake: 157 ± 125 g
	61 females						Hunger: 35.0 ± 27.0 mm
	Aged 24 ± 7 years						Slow condition
	BMI: 21 ± 1.7 kg/m^2^						ER: 21 ± 10 g/min
							Intake: 148 ± 121 g
							Hunger: 35.0 ± 27.0 mm
							No significant differences between fast and slow condition
Llewellyn 2008 [12]	N = 254 twin children	Ad libitum meal	24 sandwich quarters and chopped fruit salad	no	no	Not measured	Significant differences between groups for ER
	Overweight or obese: 68	Heritability of ER					Overweight or obese
	Higher normal weight: 87	Video					ER: 4.3 ± 0.16 bites/min
	Lower normal weight: 99	Recording of ER at home					Higher normal weight
	Monozygotic: 126						ER: 4.1 ± 0.14 bites/min
	Dizygotic: 128						Lower normal weight
	Age: 11.2 ± 0.55 years						ER: 3.8 ± 0.14 bites/min
							Heritability of ER:
							Higher for MZ twin pair than for DZ twin pairs
Andrade 2008 [14]	N = 30 healthy females	Ad libitum meals (two) eaten fast and slow	Standardized breakfast (400 kcal)	No	Type: VAS	Not measured	Meal duration: approximately 21 min longer in slow condition
	Age: 22.9 ± 7.1 years	Separate sessions	Test meal: ad libitum pasta (600 g) after 4h fast		Time: every 5 min up to 30 min and at 45 and 60 min		Fast condition
	BMI: 22.1 ± 2.9 kg/m^2^		Fast condition: with a large spoon, with no pause between bites				ER: 84.8 ± 36.32 kcal/min
			Slow condition: with a small spoon, between bites spoons were down and every bite was chewed 20–30 times				Energy intake: 645.7 ± 155.9 kcal
							Slow condition
							ER: 21.0 ± 7.2 kcal/min
							Energy intake: 579.0 ± 154.7 kcal
							Slow rates of ingestion led to significant decreases in energy intake
Kokkinos 2010 [15]	N = 17 healthy males	Fixed meals (two) within 5 min and 30 min	Test meal: ice cream 300 ML (675 kcal)	no	Type: VAS	30-min meal AUCs for PYY and GLP-1 were significantly greater than 5-min meal	30-min meal: higher fullness VAS rating immediately after the end of the meal
	Age: 29.7 ± 1.2 years	Separate	Meal duration		Time: before the test meal and at 30, 60, 90, 120, 150, 180 and 210 min after the consumption	30-min meal: PYY concentrations were higher at 90, 120, 150 min postprandially	No differences in hunger VAS ratings
	BMI: 26.1 ± 0.9 kg/m^2^	Sessions	5 min meal: 2 portions 5 min apart			No differences for ghrelin glucose and insulin AUCs values	
			30-min meal: 7 portions 5 min apart				
Zandian 2009 [16]	N = 47 healthy females	Ad libitum meals (five)	Test meal: rice, sliced chicken and vegetables (400 kj/100 g)	no	Type: VAS	Not measured	Between group comparison: Decelerated eaters ate significantly less food than linear eaters when the meal was short, interrupted and ER was increased
	17 decelerated eaters	Mandometer	control		Time: satiety ratings before the test meal and every minute after		Decelerated eaters reached a significant higher level of satiety compared to linear eaters only under the control condition
	30 linear eaters	Software records the amount of food consumed and the duration of the meal	short (40% less time of control time)		Desire to eat and hunger ratings were measured before and after the test meal		
	BMI: 22.2 (20.2–24.3) kg/m^2^		fast ER: +40% more food				
	Age: 21.2 (19.5–23.1) years		slow ER: −30% less food				
			Interrupted (1 min break every 60 g)				
Wilkinson 2016 [17]	N = 80 healthy	Eight different test meals, one for every group	Tomato soup (39 kcal/100 g)	no	Type: VAS	Not measured	Participants who saw 300 ML but actually consumed 500 ML ate at a significantly faster rate than participants who saw 500 ML but consumed 300 ml
	50 females		Custard (77 kcal/100 g)		Time: at the beginning of the test meal, immediately after eating, 20, 40, 60 min after meal termination		
	30 males		Manipulation of visual information about the amount of food, i.e., saw 300 ML but consumed 500 ml				
	8 groups of 10 participants						
	Age: 24.8 ± 8.7 years						
	BMI: 23.2 ± 3.8 kg/m^2^						
		
Henry 2018 [18]	N = 272	Video recording of eating behavioral habits	Standardized breakfast: orange juice and 2 slices of white bread with kaya spread	Ad libitum buffet: 1000 g (189 kcal/100 g)	no	Not measured	Positive association between BMR and ER, that was independent of BMI
	91 males	BMR measurements		of olive vegetable fried rice in 15 min			Positive association between ER and FFM
	Age: 40.8 ± 14.3 years						
	BMI: 23.3 ± 2.7 kg/m^2^						
	181 females						
	Age: 38.7 ± 13.8 years						
	BMI:21.6 ± 3.3 kg/m^2^						
Hawton 2018 [20]	N = 21 healthy	Normal (6 min) and slow (24 min) rate groups	Test meal: macaroni and cheese (600 kcal)	Ad libitum snacks 3h postmeal: 500 kcal crisps and 500 kcal cookies	Type: VAS	PYY: increased more in the normal rate group	Slow rate group consumed a smaller quantity of the ad libitum meal
	11 males	fMRI 2-hours postmeal while undergoing a memory task concerning the meal	Normal rate: 2 pieces every 12 sec vs. slow rate 1 piece every 24 sec		Time: at the beginning of the test meal and every 30 min for 3h	Ghrelin: suppression was greater in the slow rate group	fMRI: the slower eating group reported more accurate portion size memory
	10 females						
	Normal rate group						
	Age: 23.4 ± 4.7 years						
	BMI: 21.8. ± 2.0 kg/m^2^						
	Slow rate group						
	Age: 22.7 ± 3.3 years						
	BMI: 21.4 ± 1.7 kg/m^2^						
Forde 2013 [21]	N = 157 healthy	Four ad libitum different test meals that were modified in the texture of the meal components and the taste of the gravy	Meat, potato and vegetables (1250 kcal)	no	Type: VAS	Not measured	The ad libitum consumption in the savory mashed meal was significantly higher
	76 males		Fast condition: Savory mashed (*n* = 39)		Time: at the beginning and at the end of the meal		ER was increased in the mashed texture condition
	81 females		Standard mashed (*n* = 37)				
	Age: 44.8 ± 5.3 years		Slow condition:				
	BMI: 22.6 ± 1.7 kg/m^2^		Savory whole (*n*= 41)				
			Standard whole (*n* = 40)				
Bolhuis 2014 [22]	N = 50 healthy 11 males	Two separate days for lunch and dinner on the same day	Ad libitum lunch: 4 (700 g) hamburgers and 600 g of rice salad	Ad libitum dinner chicken noodles (463 kJ/100 g)	Type: VAS	Not measured	Compared with softer foods, lunch with harder foods led to 16% lower intake
	39 females	Video recording for oral processing characteristics	Soft–hard manipulation was established by changing the type of bread, rice and boiled vs. raw vegetables	Women were served 800 g and men 1000 g	Time: before and after ad libitum intake of the lunch and dinner		ER of the lunch with the hard foods was ~32% lower
	Age: 24 ± 2 years						Energy intake at dinner was not different after both test meals
	BMI: 21 ± 2 kg/m^2^						Oral processing data: the hard foods were consumed with smaller bites, longer oral duration per gram food and more chews per gram food
Karl 2013 [23]	N = 20 healthy	Ad libitum breakfast and lunch on the same day	Ad libitum breakfast: HED, (1.6 kcal/g ) and LED (1.2 kcal/g ) oatmeal consumed slowly (20 g/min) and quickly (80 g/min)	Ad libitum lunch 3 h after breakfast	Type: SLIM	Main effects of ED and ER on insulin, PYY, and GLP-1 AUC were observed, FR and HED being associated with larger AUC	Energy intake was higher during FR-HED
	12 males	Four separate sessions		Lasagna 1.4 kcal/g	Time: before breakfast, at 15, 30, 45, 60, 90, 120 and 180 min	No effects on active or total ghrelin AUC were documented	AUC of appetite ratings was not different between meals
	8 females	Mandometer: constant ER by following a preprogrammed eating curve on a screen					Total energy intake over both meals was higher during the FR-HED trial
	Age: 30 ± 11 years						
	BMI: 24 ± 2 kg/m^2^						
Zijlstra 2008 [24]	In real-life setting:	Study 1	Standardization of satiety before ad libitum intake Preload: mini pizza (1130 kJ)	Study 1: ad libitum test meal of liquid chocolate milk, semiliquid chocolate custard and	Type: VAS	Not measured	Study 1: the intake of the liquid was respectively 14 and 30% higher compared to the semiliquid and semisolid product
	N = 108	ad libitum intake in a real-life setting (cinema)	One-sixth of daily energy estimated needs was provided	semisolid chocolate custard	Time: before and after ad libitum intake		Study 2: in the free ER/no effort condition, the intake of the liquid was 29% higher compared to the semiliquid
	36 males	Each subject participated in three sessions	Study 1: 7 subjects received 1 mini pizza, 78 received 1.5 mini pizzas and 23 received 2 mini pizzas	Study 2			In the fixed ER/no effort condition, the intake of the liquid was 12% higher compared to the semiliquid
	72 females	Study 2	Study 2: 4 subjects received 1 mini pizza, 37 received 1.5 mini pizzas and 8 received 2 mini pizza	Liquid chocolate milk and semisolid chocolate custard			If not controlled, the difference in intake between liquid and semisolid was comparable to the real-life setting
	Age: 26 ± 7 years	ad libitum intake in laboratory setting: subjects returned for six sessions		Three conditions: Free ER, different effort			
	BMI: 22.7 ± 2.4 kg/m^2^	Test products:		Free ER, no effort			
	In laboratory setting:	Different in viscosity and equal in ED, volume and macronutrient composition		Fixed ER, no effort			
	N = 49						
	14 males						
	35 females						
	Age: 24 ± 6 years						
	BMI: 22.2 ± 2.3 kg/m^2^						
Juvonen 2009 [25]	N = 20	Two test meals with different viscosity	Isocaloric oat bran 300 ML	Ad libitum meal 3h later consisted of vegetable soup oat and rye breads, margarine, cheese, tomato, cucumber slices, noncaloric juice and tap water	Type: VAS	The beverage with low viscosity induced a greater postprandial increase in plasma glucose, insulin, cholecystokinin, GLP-1, and PYY and a greater decrease in postprandial ghrelin than the beverage with high-viscosity	Energy intake at the meal consumed ad libitum was not affected by the test beverages
	16 females	Lower viscosity produced by the modification of content of b-glucan	(1250 kJ) with low or high viscosity		Time: before the meal and at 15, 30, 45, 60, 90, 120 and 180 min		Low viscosity beverage induced a greater postprandial increase in satiety
	4 males	OGTT (75 g glucose) to ascertain normal glucose tolerance					
	Age: 22.6 ± 0.7 years	Paracetamol absorption test for gastric emptying					
	BMI: 21.6 ± 0.3 kg/m^2^						
Viskaal-van Dongen 2011 [26]	N = 37	Each subject tested a total of 7 food items (2 of them were similar for all reference foods) in separate test sessions	Measuring ingestion time: 50 g of the food with no pausing between bites or sips and eating time was recorded	Measuring ad libitum food intake: the same food in a large preweighed amount until comfortably full	Type: VAS 9-point scale	Not measured	ER ranging from 4.2 ± 3.7 to 631 ± 507 g/min
	13 males	The sample consisted of 45 food items which were tested by at least 3 subjects and a maximum of 6 subjects			Time: before and after each session		ER was positively associated with energy intake and inversely associated with ED
	24 females	Reference food tested 37 times					Carbohydrate, protein, and fiber content were inversely associated with ER in contrast to fat which showed no association
	Age: 23.3 ± 3.4 years						
	BMI: 21.7 ± 1.7 kg/m^2^						
Zhu 2013 [28]	N = 21 healthy males	Preliminary session to determine a suitable portion size for all participants	Test meal: pizza (490 kcal) into 24 portions of 3.8 × 2.5 cm	Ad libitum pasta meal 3h after the pizza (900 kcal)	Type: VAS	Plasma concentrations of glucose, insulin, GIP and CCK were higher and ghrelin was lower following the 40-chews meal	Increasing the number of masticatory cycles before swallowing increases satiety
	Mean age: 24 years range: 18–36 years	Two test sessions with different chewing time	Session 1: 8 min (15 chews)		Time: before the test meal and at 15, 30, 45, 60, 90, 120 and 180 min		There was no difference in food intake at the subsequent ad libitum meal after 3 h
	BMI: 24.8 kg/m^2^ range: 20.3–28.3 kg/m^2^		Session 2: 20 min (40 chews)				

Abbreviations: N: number; BMI: body mass index; VAS: visual analogue scale; ER: eating rate; MZ: monozygotic; DZ: dizygotic; AUC: area under the curve; PYY: peptide tyrosine tyrosine; GLP-1: glucagon like peptide 1; BMR: basal metabolic rate; FFM: fat-free mass; fMRI: functional magnetic resonance imaging; HED: high energy density; LED: low energy density; SLIM: satiety labeled intensity magnitude scale; ED: energy density; FR: fast rate; OGTT: oral glucose tolerance test; GIP: glucose-dependent insulinotropic polypeptide; CCK: cholecystokinin.

**Table 2:** Studies examining the effect of manipulating eating rate and mastication speed in patients with overweight/obesity.

First Author, Year, (Ref)	Participants	Study Design	Eating Rate Manipulation	Ad Libitum Meal after Standardized Meal	Hunger Measurements	Biochemical Profile and Hormones Response	Results
Koidis 2014 [32]	N = 14	Standardized breakfast and 3 h later a test meal	Standardized breakfast: blueberry muffin and orange juice (425 kcal)	no	Type: VAS	Not measured	Overweight/obese individuals ate at a faster rate compared to the normal-weight group
	9 females	Two different ER for each group	Test meal: chicken salad sandwich, a yoghurt and a blackcurrant drink (610 kcal)		Time: before test meal and at 15, 30, 45, 60, 90, 120 and 180 min		
	5 males	Two separate sessions	Fast ER group: consumption in 8 ± 3 min				
	Age: 22.1 ± 1.7 years		Slow ER group: consumption in 31 ± 10 min				
	7 normal-weight group BMI: 20.3 ± 2 kg/m^2^						
	7 overweight or obese group BMI: 31.7 ± 6.6 kg/m^2^						
Karl 2011 [9]	N = 25	Three test meals with different ER	Test meal: corned beef hash	Ad libitum meal 3h after test meal: lasagna	Type: SLIM	Postprandial glucose, insulin, PYY, and leptin were not affected by ER	Eating slowly delayed time to peak fullness, but did not alter peak fullness
	15 normal weight	Each volunteer received all three meals	Volunteers consumed 40% of their total energy expenditure		Time: before test meal and at 15, 30, 45, 60, 90, 120 and 180 min	ER altered the postprandial CCK and PP response, but no effects on AUC were observed	Ad libitum energy intake was not different between sessions
	8 males	Mandometer:	Meal duration				
	7 females	constant ER by following a preprogrammed eating curve on a screen	FM: 7 min				
	10 obese		MM: 14 min				
	8 males		SM: 28 min				
	2 females						
	Age: 30 ± 12 years						
	BMI: 27.3 ± 6.7 kg/m^2^						
Shah 2014 [33]	N = 70	Ad libitum meal at two different speeds	Test meal: Vegetable pasta	no	Type: VAS	Not measured	During the slow compared to the fast condition:
	36 females	Two separate days	Females: 900 g (1.300 kcal)		Time: before test meal and at 5, 10, 15, 20, 25, 30, 45, 60 min		Energy intake was significantly lower in normal-weight group
	34 males		Males: 1.200 g (1.734 kcal)				
	35 normal weight		Fast condition: with no pause between bites				
	Age: 33.3 ± 12.5 years		Slow condition: with pause between bites				
	BMI: 23.9 ± 2.6 kg/m^2^						
	35 Overweight or obese						
	Age: 44.1 ± 13 years						
	BMI: 31.3 ± 4.6 kg/m^2^						
Martin 2007 [34]	N=48	First meal:	Test meal: popcorn chicken (1000 g) cut into standard bite size units 8 g	no	Type: VAS	Not measured	Reduced rate and combined rate meals resulted in less food intake compared to baseline for males, but not for females
	22 males	Acclimation meal to determine ER of each participant	Baseline: mimic acclimation rate		Time: each minute during the meal (desire to eat)		
	26 females	Ad libitum meal at three different ER conditions	Reduced rate: by 50% of acclimation meal		Before and after the meal (hunger, desire to eat, fullness, prospective food consumption, thirst)		
	Age: 30.7 ± 10.2 years	Universal eating monitors to record food intake and generate cumulative food intake curves	Combined rate: acclimation rate at the first half and 50% reduced at the rest of the meal				
	BMI: 30.1 ± 2.9 kg/m^2^						
Scisco 2011 [35]	N = 30	Ad libitum test meal at three different speeds	Test meal: mini waffle 72 bite size pieces	no	Type: VAS	Not measured	Energy intake was less in the slow rate condition compared with the feedback condition
	23 females	Three separate sessions	Baseline condition		Time: before and after the test meal		
	7 males	Bite data were collected from an attached athletic wrist-band on the dominant wrist	Feedback: baseline with bite rate feedback				
	Age: 19.7 ± 3.5 years BMI: 25.04 ± 6.49 kg/m^2^		Slow bite rate: 50% slower from baseline				
Smitt 2011 [36]	N = 11	Three ad libitum test meals	Test meal: 500 g cooked pasta with pesto (820 kj/100 g)	no	Type: VAS	Not measured	Participants ate 12% less when chewing at 35 CPM compared to 10 CPM
	4 males	CPM were measured by	Session 1: Ad libitum chewing		Time: before and after the test meal		35 CPM resulted in longer meal duration, but also faster chewing (chews/sec)
	7 females	EMG	Session 2: 10 CPM				
	6 normal weight		Session 3: 35 CPM				
	BMI: 22.0 ± 2.0 kg/m^2^						
	5 obese						
	BMI: 33.6 ±2.1 kg/m^2^						
Spiegel 1993 [37]	N = 18 females	Ad libitum test meal with 5 different bite size pieces	Test meal: three bite sizes of tuna or turkey (5 g, 10 g, 15 g pieces) and two bite sizes of bagel with cream cheese (6 g and 12 g pieces)	no	Type: VAS	Not measured	As bite size decreased from 15 g to 5 g, the average ingestion rate decreased from 19.4 ± 2.0 to 15.9 ± 2 g/min
	9 normal weight	Five separate sessions			Time: before and after the test meal		The initial ingestion rate was decreased from 30.0 ± 2.9 to 19.6 ± 1.7 g/min
	Age: 25.1 ± 8.6 years	Chewing was monitored through					
	BMI: 21.1 ± 1.6 kg/m^2^	EMG					
	9 obese						
	Age: 32.4 ± 10.1 years						
	BMI: 32.6 ± 5.8 kg/m^2^						

Abbreviations: N: number; BMI: body mass index; ER: eating rate; VAS: visual analogue scale; FM: fast meal; MM: medium meal; SM: slow meal; SLIM: satiety labeled intensity magnitude scale; PYY: peptide tyrosine tyrosine; CCK: cholecystokinin; PP: pancreatic polypeptide; AUC: area under the curve; CMP: chews per mouthful; EMG: electromyography.

**Table 3:** Studies concerning patients with diabetes mellitus and the effect of eating rate and mastication on satiety, gut hormones, and glycemic response.

First Author, Year, (Ref)	Participants	Study Design	Eating Rate Manipulation	Ad Libitum Meal after Standardized Meal	Hunger Measurements	Biochemical Profile and Hormones Response	Results
Angelopoulos 2014 [42]	N = 20 overweight or obese with T2DM on metformin	Standard test meal at different rates	Test meal: 300 ML ice-cream (675 kcal)	no	Type: VAS	There were no differences in glucose, insulin, PYY, GLP-1 and ghrelin responses	The AUC for fullness was higher and the AUC for hunger was lower after the 30 min meal than after the 5 min meal
	Age: 62.6 ± 1.8 years	Two separate sessions	Meal duration		Time: before the test meal and at 30, 60, 90, 120, 150 and 180 min after the consumption		
	BMI: 30.6 ± 1.1 kg/m^2^		5 min meal: 2 equal portions, 5 min apart				
			30 min meal: 7 equal portions, 5 min apart				
Sun 2015 [44]	N=11	Six test sessions	Reference: glucose 50 g	no	no	Eating with chopsticks resulted in decreased postprandial glucose response	Eating with chopsticks resulted in higher chewing rate, smaller bite size, smaller number of chews per mouthful and lowered ER
	7 males	Three for glucose reference and three for different eating methods	Test meal: white boiled rice (63.6 g prior to cooking)				
	Age: 23.0 ± 0.3 years	Mastication parameters were measured by EMG	Three eating methods: chopsticks, spoon, fingers				
	BMI: 21.8 ± 0.92 kg/m^2^						
	4 females						
	Age: 24.8 ± 1.5 years						
	BMI: 19.0 ± 0.7 kg/m^2^						
Ranawana 2011 [45]	N = 12 males	Sodium acetate labeled with ^13^C was used to measure gastric emptying and breath samples were obtained every 15 min from the commencement of the meal until 240 min afterward	Test meal within 15 min	no	no	The total IAUCs for glucose and insulin were greater in the test meal with the small particles than those with the large particles	The small particles had a significant shorter gastric emptying time for T_lat_, T_lag_, T_half_, but no for T_acs_
	Age: 27 ± 5 years		Basmati rice: large and small particles				
	BMI: 23.3 ± 0.6 kg/m^2^		Participants were instructed to swallow the foods without chewing				
Ranawana 2014 [46]	N = 15	Five test sessions	Test meal: Jasmine rice within 15 min	no	no	The glucose was significantly lower when the rice was chewed 15 times than when it was chewed 30 times	
	8 males	Three to test a standard 50 g oral bolus of glucose	Session 1: 15 chews				
	7 females	Two test meals with rice	Session 2: 30 chews				
	Age: 26 ± 6 years	Mastication parameters were measured by EMG					
	BMI: 20.5 ± 4 kg/m^2^						

Abbreviations: N: number; T2DM: type 2 diabetes mellitus; BMI: body mass index; VAS: visual analogue scale; PYY: peptide tyrosine tyrosine; GLP-1: glucagon like peptide 1; AUC: area under the curve; EMG: electromyography; ER: eating rate; IAUC: incremental area under the curve; T_lat_; latency phase is the point of intersection of the tangent at the inflection point of the ^13^CO_2_ excretion curve; T_lag_: lag phase is the time taken to maximal rate of ^13^CO_2_ excretion; T_half_: half time is the time it takes 50% of the ^13^C dose to be excreted; T_acs_: ascension time is the time course between T_lat_ and T_half_ representing a period of high ^13^CO_2_ excretion rates.
